# Non-Invasive Prenatal Diagnosis of Lethal Skeletal Dysplasia by Targeted Capture Sequencing of Maternal Plasma

**DOI:** 10.1371/journal.pone.0159355

**Published:** 2016-07-19

**Authors:** Shan Dan, Yuan Yuan, Yaoshen Wang, Chao Chen, Changxin Gao, Song Yu, Yan Liu, Wei Song, Asan, Hongmei Zhu, Ling Yang, Hongmei Deng, Yue Su, Xin Yi

**Affiliations:** 1 Department of Perinatal Medicine, Beijing Obstetrics and Gynecology Hospital, Capital Medical University, Beijing, China; 2 Binhai Genomics Institute, BGI-Tianjin, BGI-Shenzhen, Tianjin, China; 3 Tianjin Translational Genomics Centre, BGI-Tianjin, BGI-Shenzhen, Tianjin, China; 4 BGI-Shenzhen, Shenzhen, China; 5 Guangzhou Key Laboratory of Cancer Trans-Omics Research (GZ2012, NO348), BGI-Guangzhou, BGI-Shenzhen, Guangzhou, China; National Cheng Kung University Medical College, TAIWAN

## Abstract

**Background:**

Since the discovery of cell-free foetal DNA in the plasma of pregnant women, many non-invasive prenatal testing assays have been developed. In the area of skeletal dysplasia diagnosis, some PCR-based non-invasive prenatal testing assays have been developed to facilitate the ultrasound diagnosis of skeletal dysplasias that are caused by de novo mutations. However, skeletal dysplasias are a group of heterogeneous genetic diseases, the PCR-based method is hard to detect multiple gene or loci simultaneously, and the diagnosis rate is highly dependent on the accuracy of the ultrasound diagnosis. In this study, we investigated the feasibility of using targeted capture sequencing to detect foetal de novo pathogenic mutations responsible for skeletal dysplasia.

**Methodology/Principal Findings:**

Three families whose foetuses were affected by skeletal dysplasia and two control families whose foetuses were affected by other single gene diseases were included in this study. Sixteen genes related to some common lethal skeletal dysplasias were selected for analysis, and probes were designed to capture the coding regions of these genes. Targeted capture sequencing was performed on the maternal plasma DNA, the maternal genomic DNA, and the paternal genomic DNA. The de novo pathogenic variants in the plasma DNA data were identified using a bioinformatical process developed for low frequency mutation detection and a strict variant interpretation strategy. The causal variants could be specifically identified in the plasma, and the results were identical to those obtained by sequencing amniotic fluid samples. Furthermore, a mean of 97% foetal specific alleles, which are alleles that are not shared by maternal genomic DNA and amniotic fluid DNA, were identified successfully in plasma samples.

**Conclusions/Significance:**

Our study shows that capture sequencing of maternal plasma DNA can be used to non-invasive detection of de novo pathogenic variants. This method has the potential to be used to facilitate the prenatal diagnosis of skeletal dysplasia.

## Introduction

Skeletal dysplasias are a group of heterogeneous genetic diseases that affect the development of the chondro-osseous tissue and that have an estimated prevalence of 2-5/10000 individuals at birth [1]. Two-dimensional ultrasound is widely used in prenatal detection of skeletal dysplasia; however, the sensitivity of this method is approximately 40–60% [2]. A clear diagnosis that specifically differentiates lethal dysplasia from non-lethal dysplasia is important for prognosis, proper management and genetic counselling. Targeted molecular analysis of foetal DNA can be used in facilitating a definitive diagnosis because an increasing number of mutations responsible for skeletal dysplasia have been identified. The foetal genetic material needed for molecular testing is obtained traditionally using invasive sampling procedures such as amniocentesis or chorionic villus sampling. However, these invasive sampling procedures have a risk of miscarriage or infection.

Since the discovery of cell-free foetal DNA in the plasma of pregnant women, many non-invasive prenatal testing assays have been developed, such as non-invasive foetal chromosomal abnormality detection [3–6] and non-invasive prenatal diagnosis of single-gene diseases [7–9]. Concerning skeletal dysplasias, some assays have been developed for non-invasive detection of foetal de novo mutations responsible for achondroplasia [10–12], thanatophoric dysplasia [13], and others. However, most of these assays are based on PCR, which can only detect limited loci simultaneously, and an accurate sonographic diagnosis is needed to narrow down the candidate target mutations. However, the sonographic misdiagnosis of skeletal dysplasia is common due to the variability and overlapped features in this group of disorders [1]. Thus, the detection rate is dependent primarily on the doctor's proficiency and professionalism, and the definitive diagnostic rate of these non-invasive methods is limited. Development of targeted capture sequencing technology allows the simultaneous analysis of many candidate genes or targets and is a helpful tool for differential and definitive diagnosis, particularly when a diagnosis based on phenotype is suspected. Recently, methods based on target region capture sequencing have been developed successfully for the detection of ultra-low frequency mutations in cell-free plasma DNA and have been widely used in cancer research [14, 15]. Kitzman et al [16] also reported the importance and the feasibility of non-invasive de novo mutation detection in maternal plasma DNA by massive parallel sequencing. However, this method requires whole genome sequencing, which may not be suitable for clinical application. In this study, we present a novel strategy based on targeted capture sequencing to realize non-invasive foetal de novo mutation detection and to facilitate the differentiated diagnosis of skeletal dysplasias. We recruited 3 skeletal dysplasia-affected families for feasibility tests using a sensitive, low frequency mutation detection algorithm and an established process to annotate and review the pathogenic mutation, and the de novo pathogenic mutations in these 3 cases were all identified successfully.

## Materials and Methods

### Sample collection

Three pregnant women who were referred for antenatal ultrasound diagnosis at the Beijing Obstetrics and Gynaecology Hospital, Capital Medical University, were recruited for this study. This study was approved by the Ethics Committee of Beijing Obstetrics and Gynaecology Hospital, Capital Medical University, and written informed consent was obtained from each participant.

All the pregnant women and their husbands were normal; however, their foetuses were found to be affected by skeletal dysplasia during the prenatal ultrasound screening. Before amniocentesis, 5 ml EDTA anti-coagulated blood was drawn from each pregnant woman and from her husband. Maternal plasma was separated within 8 h by an initial centrifugation at 1,600×g for 10 min, followed by centrifugation at 16,000×g for 10 min. In total, 20 ml amniotic fluid was collected for molecular diagnosis. Plasma samples were stored at -80°C, and other samples were stored at -20°C. Two cases whose foetuses were not affected by skeletal dysplasia were also included as negative controls.

### Assay design

To develop a non-invasive prenatal testing assay that can facilitate the diagnosis of lethal skeletal dysplasia, 16 genes associated with some important lethal skeletal dysplasias were selected for analysis (Table 1) [17]. Probes were designed to capture the coding regions of these genes of interest and the 10 bp flanking the coding regions. The targeted region is approximately 1.2 Mb and contains other genes related to short stature.

**Table 1:** List of the selected lethal skeletal dysplasias.

Disease	Gene	Inheritance
Achondrogenesis Type IA	TRIP11	AR
Achondrogenesis Type IB	SLC26A2	AR
Achondrogenesis Type II or Hypochondrogenesis	COL2A1	AD
Thanatophoric Dysplasia Types I and II	FGFR3	AD
Short Rib-Polydactyly Syndrome Types I, IIB, and III	DYNC2H1	AR
Short Rib-Polydactyly Syndrome Type IIA	NEK1	AR
Fibrochondrogenesis Type I	COL11A1	AR
Atelosteogenesis Types I and III	FLNB	AD
Atelosteogenesis Type 2	SLC26A2	AR
Perinatal Osteogenesis Imperfecta	COL1A1, COL1A2, CRTAP, LEPRE1, PPIB, BMP1	AD, AR
Hypophosphatasia	ALPL	AD, AR

### Target region capture sequencing

Cell-free DNA was extracted from 0.6–1.8 ml plasma using a QIAamp Circulating Nucleic Acid Kit (Qiagen). The plasma DNA libraries were prepared using a KAPA Library Preparation Kit (KAPA Biosystems) according to the manufacturer’s protocol without DNA shearing. After the adapter was ligated, 8 cycles of PCR were performed with index primers to barcode different samples. Genomic DNA was extracted from the amniotic fluid or blood using a QIAamp DNA Mini Kit. Briefly, 1 μg genomic DNA was sheared into small fragments with a predominance of 200–250 bp. Then, after the samples underwent end repair, overhanging A addition and adapter ligation, 4–5 cycles of PCR were performed with index primers to barcode different samples. The maternal plasma DNA and the genomic DNA of the parents were captured by the same set of custom-designed NimbleGen SeqCap EZ probes, followed by paired-end sequencing (101 bp read) using a HiSeq 2500 system.

### Detection of single nucleotide variants

The paired-end sequencing reads were aligned to the human reference genome (Hg19, GRCh37) with BWA MEM (0.7.12) [18] using the default parameters. For parental and foetal genomic DNA samples, the variants were called by GATK[19]. For maternal plasma DNA samples, a modified pipeline was used to detect variants with minor allele frequency (MAF)>1%. Only single nucleotide variants were considered in this study. Briefly, Picard (1.87) [20] was used to mark the PCR duplication reads and to fix the mate information. Low quality reads with base error rate or mapping error rate larger than 5% were discarded. Then, a Bayesian methodology described by Frampton et al [21] was used to call base substitution,and a base substitution would be called if the probability of being sequencing error was lower than 1%. Fisher’s test was used to filter the strand bias, and the Kolmogorov-Smirnov test was used to filter the read location bias (P<1e-6). Then error SNV calls caused by small indels were filtered out using CIGAR information [22]. Schmitt et al [23] have proved that the PCR or sequencing errors could be corrected using sequence information of the PCR duplication and two strands of a DNA duplex. Thus, the calls were further filtered by duplication reads and sequence information of complementary strands, only variants supported by at least two duplication cluster, with each cluster containing at least 3 reads and all of the reads yielding the same sequence at the position, or variants that were supported by two complementary stands of a DNA duplex were further analysed.

### Estimation of the fraction of foetal DNA in maternal plasma

The foetal DNA fraction was calculated to confirm the presence of the foetal DNA in the maternal plasma cell-free DNA. This fraction was calculated according to the following formula:

$$\text{feotal DNA fraction}=\frac{d_{foetus}}{\left(d_{mother}+d_{feotues}\right)}$$

where *d*_*mother*_ stands for the counts of the alleles shared by the mother and the foetus, and *d*_foetus_ stands for the counts of foetal specific alleles [24].

### Assessment of foetal specific base substitution detection performance

We defined a SNP as foetal specific if it is present in maternal plasma DNA or foetal gDNA, but absent in the maternal gDNA. Foetal specific variant could be determined using SNP site that is heterozygous in foetus (e.g., CT) but homozygous in mother (e.g., TT). In this situation, C allele is foetal specific allele. Because de novo mutation is a special type of foetal specific variant, the performance of foetal de novo variant could be represented by that of foetal specific variant. Thus, we evaluated the performance of our method by comparing the non-reference foetal specific variants identified in plasma sample with that identified in paried foetal gDNA samples, and the results of foetal gDNA sample were considered the standard. To minimize the interference of low quality SNPs, we filtered out sites located in repeat sequence region, covered by less than 20 reads in either foetal gDNA sample or maternal gDNA sample, and sites where identified as homozygous but contain discordant sequence in more than two reads in gDNA sample. To increase the number of variants used for evaluation, data of the whole gene panel, in addition to the selected 16 genes were used. Only single nucleotide variants in coding regions were involved in the analysis. The true positive rate (TPR) and positive predictive value (PPV) of our method were calculated according to the following formulas:

$$\begin{array}{l} TPR=\frac{TP}{TP+FN}\\ PPV=\frac{TP}{TP+FP} \end{array}$$

Where *TP* stands for the number of true positive variants that were shared by the plasma DNA sample and foetal gDNA sample, *FN* stands for the number of false negative variants that were detected in the foetal gDNA sample but absent in the plasma DNA sample, and *FP* stands for the number of false positive variants that were detected in the plasma DNA sample but absent in the foetal gDNA sample.

Sequencing error rate was calculated using SNPs that were same homozygous genotype in both the amniotic fluid DNA and paried maternal genomic DNA according to the formula:

$$\text{Error rate}=\frac{N_{err}}{N}$$

where *N*_*err*_ stands for the counts of error bases and *N* stands for the total counts of all bases.

### Identification of foetal de novo mutations in maternal plasma cfDNA

We used a self-developed pipeline to identify de novo mutations in plasma DNA samples or foetal genomic DNA samples. A list of non-reference alleles was generated from the VCF file of each plasma DNA sample or foetal genomic DNA sample. Then, SAMtools mpileup [22] was used to extract genotype information regarding each locus on the list generated from the parent data, the maternal plasma DNA or foetal genomic DNA for de novo variant identification. For parental and foetal genomic DNA samples, variants with allele frequency range from 0.25 to 0.75 were classified as heterozygous. To minimize the false positive rate, we further filtered out variants located in tandem repeats or segmental duplication sequences, regions covered by less than 20 reads in any of the three samples, and variants that also identified in parents with more than two reads or allele frequency larger than 1%.

### Variant interpretation strategy

Because no phenotypic abnormalities of the parents or family histories were reported in these families, we attempted to find de novo dominant pathogenic variants present in plasma DNA samples. Only variants in the coding sequence were analysed. If no de novo dominant variant was founded in the plasma DNA samples, then the possibility of recessive inheritance was further considered. The parental data were used to check the inheritance mode of the candidate variants. Plasma samples and foetal genomic DNA samples were analysed, and the results were compared to evaluate the accuracy of this non-invasive method.

**Fig 1:**
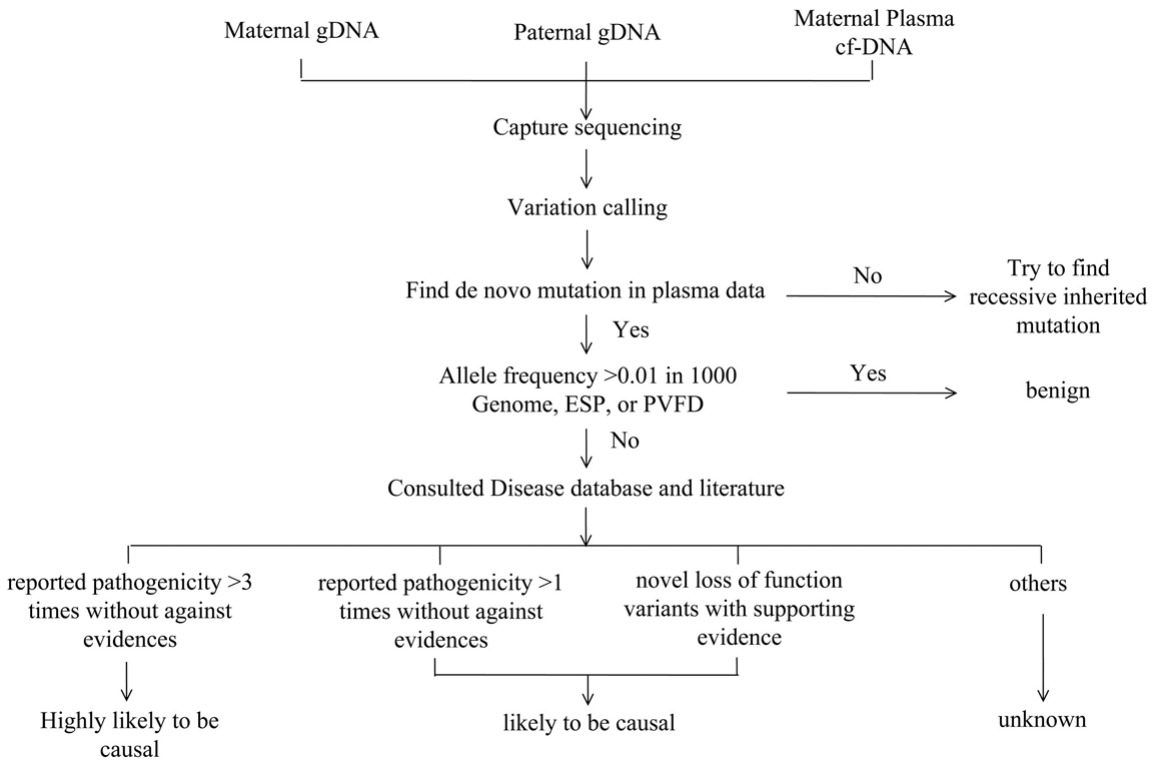
Strategy of variant interpretation.

The strategy used for variant interpretation is illustrated in Fig 1. Because the estimated prevalence of skeletal dysplasia is approximately 2–5/10000 individuals at birth [1], we reasoned that a single allele with frequency >0.01 in large population studies is not pathogenic. Variants with allele frequency >0.01 in any of the population data (from the 1000 Genome Project, NHLBI Exome Sequencing Project (ESP), ExAC, and BGI Database [PVFD]) were classified as likely benign. The pathogenicity of each remaining variant was determined by systemic disease database consulting (LOVD, HGMD, and others) and literature searching. Three types of variants could be reported: 1) pathogenic variants that have been reported as pathogenic multiple times without opposing evidence, 2) likely pathogenic variants that have been reported as pathogenic with supporting evidence at least once and without opposing evidence, and 3) novel loss of function variants (i.e., nonsense, frameshift or splice site) with supporting evidence (absent in controls, evidence of segregation, or similar types of pathogenic mutations have been reported for the gene).

### Computer simulation

We used computer simulation to investigate the sensitivity and specificity of our method for foetal specific variant detection in different foetal DNA concentration. We built the statistical model based on the assumption that the sequencing depth, sequencing error, counts of foetal specific subsititution bases and the duplicated reads for each allele should follow a poisson distribution. Assuming the mean depth of coverage of the plasma sample is detph_mean, and the foetal DNA concentration is foetal_conc, then the sequencing depth of a given site (S), the expected sequencing depth of foetal-specific allele (S_foetal_), and the sequencing error (S_err_) could be simulated using possion distribution:

S ~ Pois (λ=depth_mean)

$${\text{S}}_{\text{foetal}}\;\sim \text{ Pois}(\lambda =\text{depth}\_\text{fetal}\_\text{mean})$$

$${\text{S}}_{\text{err}}\;\sim \text{ Pios}(\lambda =\text{depth}\_\text{err}\_\text{mean})$$

respectively, where depth_err_mean stands for the expected sequencing error and was calculated as

$$\text{d}epth\_err\_mean=depth\_mean\times P_{err}$$

and *P*_*err*_ was stands for mean sequencing error rate which was calculated from the real samples.

The probability of one read is support by at least 3 duplication was calculate as

$$\text{P}\_\text{dup}={\text{P}}_{\text{Poisson}}\text{(X}\geq 3|\lambda =\text{dup}\_\text{mean})$$

Then we assumed that the probability of observing a foetal-specific variant is supported by at least 2 duplication clusters and each cluster contains at least 3 PCR duplicated reads should follow a binomial distribution and could be calculated as

$$\text{P}=\text{P(X}\geq 2|\text{n}={\text{S}}_{\text{foetal}},\text{ p}=\text{P}\_\text{dup})$$

For illustration, we assumed the duplication rate is 50%. Based on these assumptions, we simulated 10^5^ maternal homozygous sites with foetal specific variant and 10^8^ maternal homozygous sites without fetal specific variant for different fetal DNA concentration and sequence depth to investigate the detection rate of foetal specific variants and miscalling of sequencing errors, and the TPR and PPV were calculated.

## Results

### Target region capture sequencing

Three families whose foetuses were observed to have skeletal abnormalities and two families affected with other single gene diseases were recruited for this study. Targeted capture sequencing was performed on maternal plasma DNA and the parental genomic DNA. Amniotic fluid samples were also sequenced as controls. For the plasma DNA samples, the mean depth of the targeted region was approximately 285.17 after the duplicate reads were filtered (range, 141.20–359.62). A mean of 98.34% of the region was coverage by at least 20 reads. The mean duplication rate of plasma DNA sample was 55.07% (range, 49.74%-65.16%). The mean depth of the gDNA samples was approximately 234.54 after the duplicate reads were filtered (range, 143.04–300.27). A mean of 96.4% of the region was coverage by at least 20 reads. The mean duplication rate of gDNA sample was 18.59% (range, 8.99%-25.19%). The mean sequencing error rate was 0.092% (range: 0.057%-0.176%) (Table 2).

**Table 2:** Basic statistic of target region capture sequencing.

Family	Sample	Reads mapped to the target region (M)	Mean depth before filtering duplicate reads	Mean depth after filtering duplicate reads	Duplication rate	>20×	Sequencing error	Mean depth of foetal specific SNV allele
Case 1	foetus	0.67	262.84	209.87	19.48%	96.93%	0.102%	-
	mother	0.54	212.57	174.34	17.43%	98.21%	0.104%	-
	father	0.54	207.77	171.41	16.86%	98.47%	0.109%	-
	plasma	2.14	853.01	141.20	53.42%	95.60%	0.059%	14.03
Case 2	foetus	0.46	163.37	143.04	11.37%	84.49%	0.086%	-
	mother	0.73	285.67	258.55	8.99%	97.66%	0.176%	-
	father	0.44	171.40	143.74	15.62%	96.32%	0.093%	-
	plasma	4.67	1846.32	293.19	65.16%	99.75%	0.089%	12.77
Case 3	foetus	0.54	214.77	174.32	17.96%	95.28%	0.096%	-
	mother	0.59	232.91	188.84	18.39%	98.04%	0.094%	-
	father	0.58	227.14	174.97	22.17%	97.71%	0.093%	-
	plasma	1.89	750.26	339.60	49.74%	98.64%	0.057%	49.69
Control Case 1	foetus	0.88	346.06	274.15	19.87%	98.37%	0.105%	-
	mother	0.48	190.31	147.33	22.13%	96.22%	0.097%	-
	father	0.74	291.73	226.29	21.95%	97.98%	0.090%	-
	plasma	2.49	988.09	292.23	51.52%	99.06%	0.063%	21.86
Control Case 2	foetus	1.05	417.44	300.27	25.19%	96.14%	0.094%	-
	mother	0.54	212.08	175.44	16.83%	96.46%	0.091%	-
	father	0.91	358.33	268.44	24.54%	98.27%	0.091%	-
	plasma	2.32	922.43	359.62	55.50%	98.78%	0.059%	15.29

Foetuses of case 1 and control case 2 were identified as male due to the high mean depth of region of interested in chromosome Y (S1 Table). For case1 and control case 1, we further analyzed the size distribution of foetal DNA and total DNA in plasma sample using reads mapped to chromosome Y and whole region, respectively. The results showed that the size distribution of foetal DNA was shifted toward the shorter end compared with the total DNA (S1 Fig).

### The performance of foetal specific base substitution detections in maternal plasma DNA

**Fig 2:**
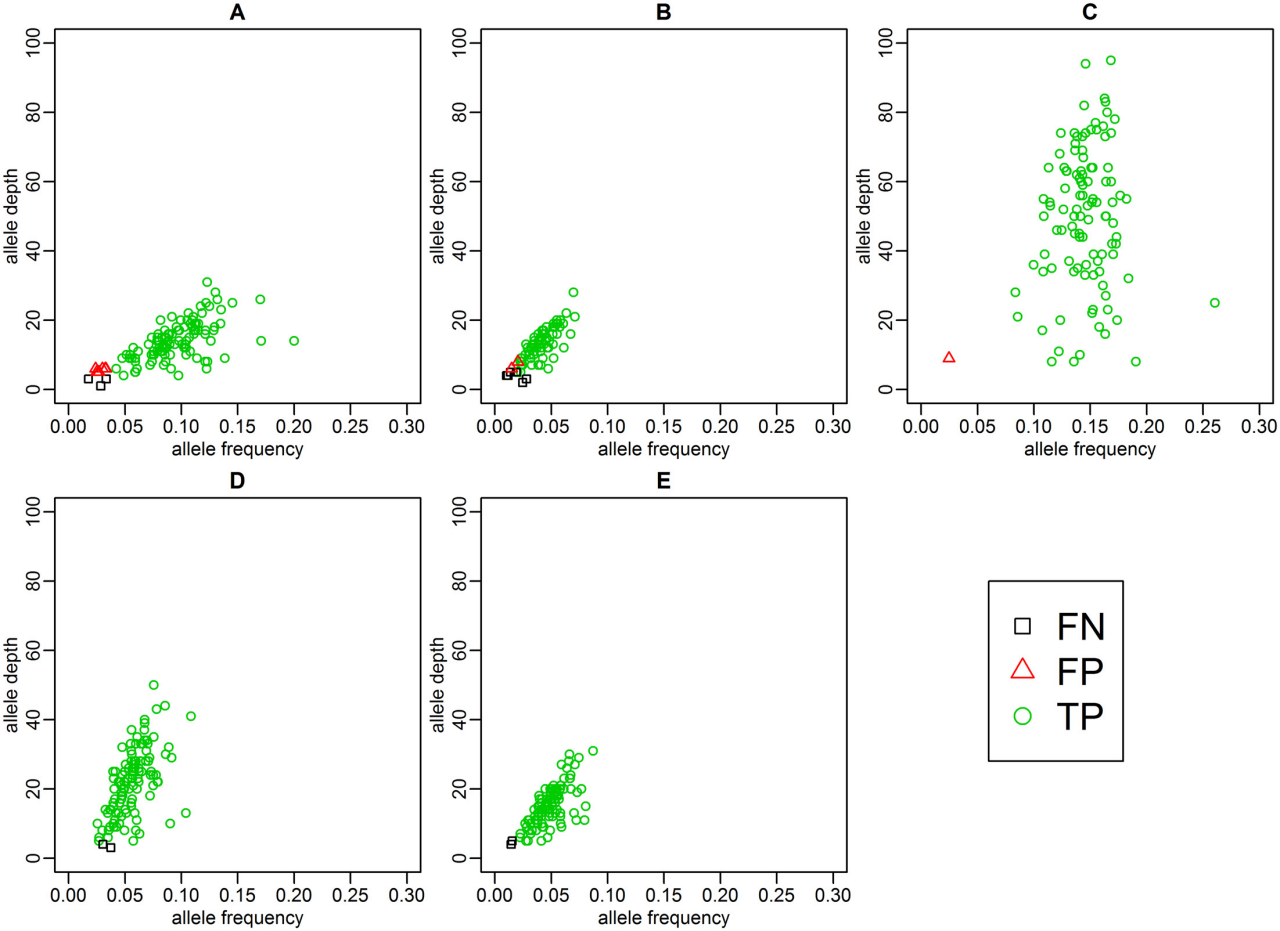
The distribution of allele frequency and allele depth of real positive foetal specific variants, false positive foetal specific variants and false negative foetal specific variants after the duplicate reads were filtered. (A) Case 1. (B) Case 2. (C) Case 3. (D) Control case 1. (E) Control case 2. The green circle represents the allele that was shared by the foetal gDNA and plasma DNA (real positive), the black square represents the allele that was detected in foetal gDNA but was not detected in plasma DNA (false negative), and the red triangle represent the allele that was only detected in plasma DNA (false positive).

An average of 105 foetal specific variants (range: 79–115) were identified in foetal gDNA samples and were used for assessing the performance of foetal specific base substitution detection in plasma samples. The foetal DNA concentration of these five cases were ranged from 6.84% to 30.56%. The results showed that an average of 97.25% variants that were detected in the foetal gDNA samples could be identified successfully in the plasma samples, with a TPR range from 92.41% to 100% (Table 3). The number of false positive alleles ranged from 2–7 in each sample with a mean PPV of 98% (range from 93.97% to 100%). We analyzed the allele depth and allele frequency distribution of TP, FP and FN in these five samples. Results showed that the false negative or false positive calls tended to occur in alleles with low supported reads or low allele frequency. Additionally, the allele frequency of most of the error calls deviated far from the foetal fraction (Fig 2). Details of the TP, FP, and FN alleles in each case could be seen in S2–S6 Tables.

**Table 3:** Performance of foetal specific base substitution detection in plasma samples.

Case No.	Foetal conc	Number of foetal specific variants identified in fetal gDNA sample	Number of FN	Number of FP	Number of TP	TPR	PPV
Case 2	6.84%	79	6	2	73	92.41%	97.33%
Control case 1	9.49%	114	2	0	112	98.25%	100.00%
Control case 2	10.37%	114	2	0	112	98.25%	100.00%
Case 1	20.76%	112	3	7	109	97.32%	93.97%
Case 3	30.56%	105	0	1	105	100.00%	99.06%

### Detection of causal variants

The clinical information and ultrasound examination of the three cases were shown in S7 Table and S2–S4 Figs, all foetus were affected with skeletal abnormality. Variants detected in the coding region of the 16 gene of interested were annotated and interpreted as described in method. De novo foetal specific variants and paternal inherited foetal specific variants were determined by comparing plasma DNA sample to the parental gDNA samples. The classification of variants detected in plasma samples were shown in Table 4. The annotation results of the five cases can be seen in S8–S12 Tables.

**Table 4:** the statistic of interpretation results of SNV detected in maternal plasma.

	SNP type	numbers	foetal specific variant		maternal non-ref-hom allele	maternal heterozygous allele
			de novo	paternal inherited non-reference allele		
case 1	total SNV	39	1	7	15	16
	Synonymous	21	-	3 benign	8 benign	10 benign
	missense	14	1 pathogenic	3 benign	6 benign	4 benign
	splice±10	4	-	1 benign	1 benign	2 benign
case 2	total SNV	51	2	4	17	29
	Synonymous	26	1 benign (false)	2 benign	9 benign	14 benign
	missense	21	1 pathogenic	1 benign	6 benign	10 benign; 3 VUS
	splice±10	5	-	-	2 benign	2 benign
case 3	total SNV	44	1	12	15	16
	Synonymous	25	-	7 benign	8 benign	9 benign, 1VUS
	missense	14	1 pathogenic	3 benign	5 benign	4 benign; 1VUS
	splice±10	5	-	2 benign	2 benign	1
Control case 1	total SNV	40	0	9	13	19
	Synonymous	20	-	5 benign	7 benign	8 benign
	missense	16	-	3 benign	4 benign	8 benign;1VUS
	splice±10	5	-	1 benign	2 benign	1 benign; 1 VUS
control case 2	total SNV	45	0	10	23	12
	Synonymous	24	-	5 benign	13 benign	6 benign
	missense	14	-	3 benign	8 benign	3 benign
	splice±10	7	-	2 benign	2 benign	3 benign

In case 1, the pregnant woman was 26 years old, gravida 2, para 0, and referred for antenatal sonographic diagnosis at 27 weeks of gestation. Ultrasound examination revealed a single live foetus with a small thoracic cage and bell-shaped long bones of the legs and arms (S2 Fig). The foetal DNA fraction of the plasma DNA was 20.76%. A total of 39 variants were detected in region of interested, among them 7 variants were paternal inherited foetal specific variants and one was de novo variant. Only the de novo variant, c.742C>T (p.R248C) in *FGFR3* (NM_000142.4), was classified as pathogenic variant. Six reads supported this variant after the duplicate reads were filtered; 19% of the total reads cover this loci. This variant is one of the most common pathogenic variants known to cause type I thanatophoric dysplasia [25,26].

In case 2, the pregnant woman was 29 years old, gravida 1, para 0, and referred for antenatal sonographic diagnosis at 22 weeks of gestation. Ultrasound examination revealed a breech, single live foetus with a deformed skull and bowed long bones of the arms and legs. The long bone measurement was less than the 5th percentile of normal biometry. The foetal size was less than the 10th percentile of normal biometry (S3 Fig). The foetal DNA fraction in plasma was 6.84%. A total of 51 variants were detected in the region of interested, among them two variants were paternal inherited foetal specific variants and two was de novo variant. One de novo variant, c.1774G>A (p.G592S) in COL1A2 (NM_000089.3), was classified as pathogenic. After the duplicate reads were filtered, 10 reads supported this variant; 4% of the total reads cover this allele. This variant has been reported to cause type II osteogenesis imperfecta [27]. The other de novo mutation was a false positive variant and it was a synonymous variants with a high population frequency. The 2 paternal inherited foetal specific variants were classified as benign, and among the 46 variants shared by mother, 3 were classified as VUS, 43 were classified as benign.

In case 3, the pregnant woman was 29 years old and referred for antenatal sonographic diagnosis at 28+2 day weeks of gestation. Ultrasound examination revealed a single live foetus. The length of its long bone was shorter than expected, and its right femur was slightly bent (S4 Fig). The foetal DNA fraction in plasma was 30.56%. A total of 44 variants were detected in the region of interest, with 12 paternal inherited foetal specific variant and one de novo mutation. The de novo mutation detected in the plasma sample, namely, c.1138G>A (p.G380R) in FGFR3 (NM_000142.4), is the major pathogenic variant known to cause achondroplasia, which corresponds to 98% of achondroplasia. After the duplicate reads were filtered, 56 reads supported this variant; 14% of the total reads cover this allele. For the remaining 43 variants, 41 were classified as benign, 2 were classified as VUS.

For the two control cases, not pathogenic mutation were detected in plasma samples in this region. These results were identical to those obtained by sequencing foetal gDNA samples.

We examined whether the three pathogenic alleles were also present in other non-affected individuals. The results showed that we could find 1–2 reads that carried the pathogenic variant in a few non-affected individuals in the bam files; however, the allele frequency is extremely low, and this noise could be easily distinguished from the real signal (S13–S15 Tables). These errors were already filtered out by our pipeline, and none of these errors was present in our final result files used for further interpretation.

### Computer simulation

**Fig 3:**
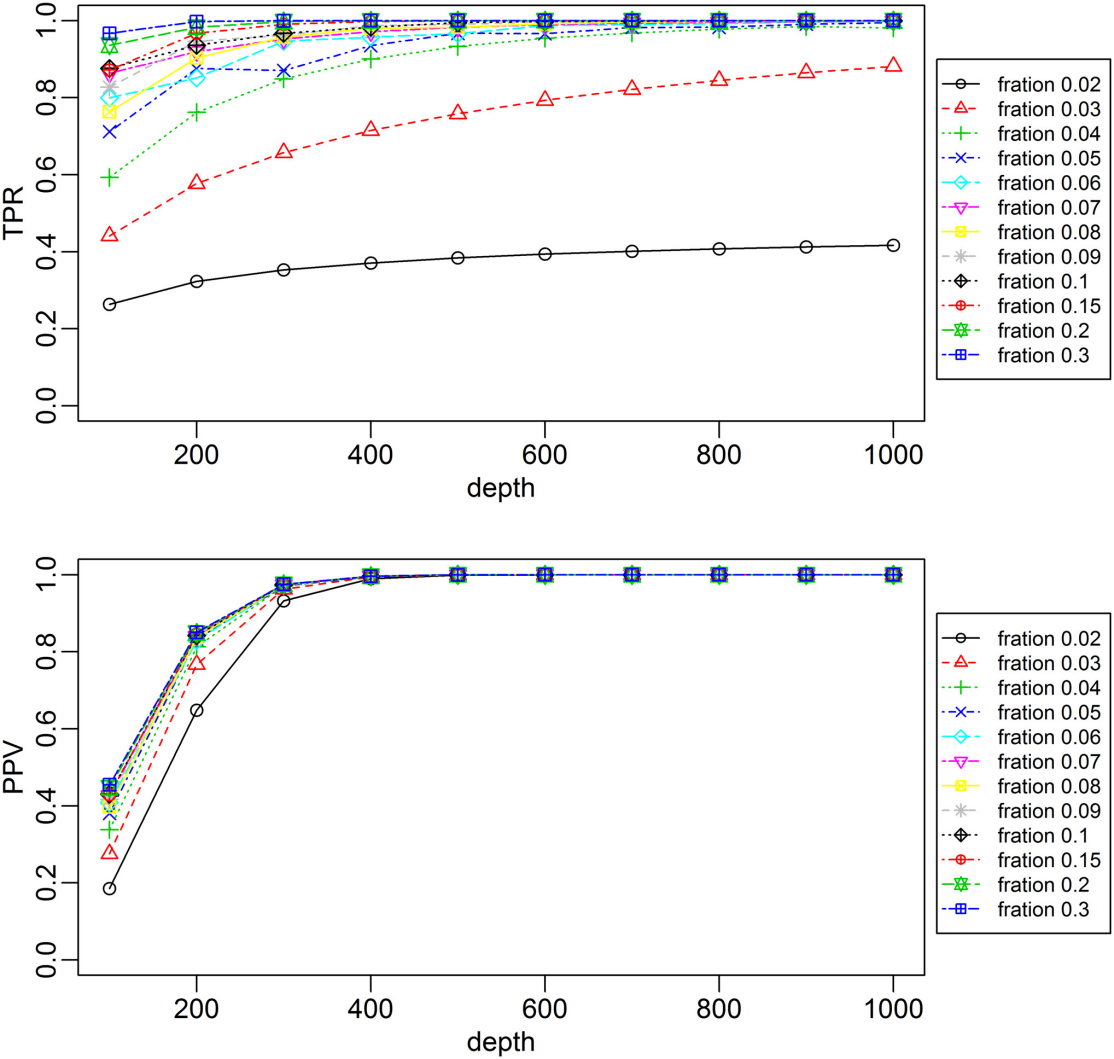
**Computer simulation of foetal specific variants detection in different foetal DNA concentrations and different mean sequencing depth** (A) postive predict value. (B) true positve rate.

Computer simulation was performed to estimate the performance of foetal specific variants detection in different foetal DNA fraction and to determine the parameter that would improve the accuracy of our method. We assumed that the duplication rate of plasma sample was 50%, then for a given foetal DNA fraction, we gradually increased the de-duplication sequencing depth from 100 to 1000 with 100 interval to investigate the accuracy for foetal specific variants detection. The results showed that increasing the sequencing depth would improve both the sensitivity and specificity of our method. If the fraction fetal DNA concentration was higher than 10%, the de-duplication sequencing depth of 430 was adequate to obtain an accurate detection (PPV>99%, PR>99%). When the fetal DNA concentration was decreased, the de-duplication sequencing depth would need to be increased to achieve the same performance. Additionally, more sequencing detph was needed to achieve the high sensitivity (TPR>99%) than high specificity (PPV>99%). For specificity, if the foetal DNA concentration decreased to 2%, a sequencing depth of 400 was adequate to obtain a positive predict value (PPV)>99%. For sensitivity, when the foetal DNA fraction was decreased to 5%, a sequencing depth of 870 would be need to achieve high sensitivity(TPR>99%), and for sample with foetal DNA fraction lower than 4%, a sequencing depth of more than 1000 would be needed (Fig 3).

## Discussion

Most of the previously reported methods for non-invasive detection of foetal specific alleles were developed based on PCR and RFLP, which rely on changes in the recognition sequence for a specific enzyme. However, a change in the enzyme recognition site may be absent in some mutations of interest, and this method is not suitable for targeting multiple regions. Unlike the PCR-based method, the targeted capture sequencing can detect many candidate genes and regions simultaneously, which may increase the diagnosis rate, particularly in some suspected cases, and can be extended easily to other genes.

Here, we investigated whether the targeted capture sequencing of maternal plasma cf-DNA could be used for non-invasive prenatal detection of foetal de novo mutations and facilitate the diagnosis of foetus skeletal dysplasia. Three pregnant women whose foetuses were affected by skeletal dysplasia were involved in our study. The causal de novo variants were identified successfully in the three plasma samples and were consistent with the invasive molecular diagnosis results. The detection of the three pathogenic variants in the plasma was specific. According to the non-invasive prenatal detection results, the foetuses of cases 1, 2, and 3 were diagnosed as type I thanatophoric dysplasia, osteogenesis imperfecta type II, and achondroplasia, respectively. The case affected by achondroplasia would have a better prognosis than the other cases, the intelligence and life span of individuals affected by achondroplasia are usually near normal. In the other two cases, the detection results indicated that the affected individuals would be lethal in the prenatal period or die shortly after birth in most situations. Our data showed that the de novo mutation can be identified easily in the plasma by our method; and that the introduction of this non-invasive molecular detection method may be helpful in improving the accurate diagnosis rate of skeletal dysplasia, which may be useful for clinical prognosis.

We observed that the allele frequencies were distributed in a relatively wide range near the estimated foetal DNA fraction (Fig 2). Using case 1 as an example, the foetal DNA fraction was calculated as 20.76%, and the reads supporting this foetal-specific variant should be approximately 10% under ideal conditions. However, 19% reads supported the variant c.742C>T (p.R248C) in FGFR3. We further analysed the maternal and paternal gDNA capture sequencing data by the same pipeline used for plasma DNA analysis, and no reads supported the detected variants; thus, the possibility of somatic mutation in maternal gDNA could be excluded, and this bias may be an experimental bias. Thus, filtering out the false positive calls by setting a threshold of allele frequency directly according to the foetal DNA fraction may not be possible. We used duplicate reads to further filter out the false positive calls. We compared the accuracy of foetal specific variants detection before (S16 Table) and after the filtration (Table 3), results showed that this method was very helpful in reducing false positive calls without changing the sensitivity.

The detection sensitivity correlated to the sequence depth and the foetal DNA fraction in each sample. The false negatives tended to occur in regions without sufficient sequence coverage (Fig 2). In case 2, the foetal fraction was approximately 6%, and the TPR of foetal non-reference-specific allele in maternal plasma was approximately 92%. In contrast, the sample whose foetal DNA fraction was higher than 10% had a TPR value higher than 97%. We performed a computer simulation experiment to study the influences of foetal DNA concentration and sequencing depth on the performance of our method. The simulation results showed that increasing the sequence depth would be a useful measurement to improve the detection sensitivity in samples with low foetal DNA concentration. If we assumed the duplication rate is 50%, then for samples with foetal DNA concentration of 5%, a deduplication sequence depth of 870 would allow the highly accurate foetal specific variant detection (PPV>99%, TPR>99%). According to Lo et al.’s study, there is approximately 1000 genome equivalents of DNA in 1 mL plasma [28], thus it is possible to reach the required sequencing depth of 870 by increasing the input of plasma sample and the sequencing data production. And 5% foetal DNA concentration seems close to the threshold detection limitation of our method, since for 4% foetal DNA concentration simulation, when the sequencing depth increased to 1000, the TPR value was still lower than 99% and had reached a plateau. Thus, calculating the foetal DNA fraction for each sample as a quality control is important.

We checked the distribution of allele frequency in three types of alleles in the five plasma samples: 1) alleles that were heterozygous in both the mother and the foetus; 2) alleles that were heterozygous in the mother, while the foetus was homozygous as the reference, or 3) alleles that were heterozygous in the mother, while the foetus was homozygous for non-reference variants. A large overlap was observed between each status, particularly when the foetal DNA fraction was low, as shown in S5 Fig. The allele ratio distribution made it impossible to determine whether the foetus inherited a causal variant from its mother. Thus, the detection of recessive causal variants in plasma might not lead to the conclusion that the foetus was affected by a certain recessive disease but may indicate a high risk of being affected by that disease, particularly when two pathogenic mutations are detected in one gene in the plasma sample and one is inherited from its father.

In conclusion, we have demonstrated that foetal de novo disease-causing variants can be identified in maternal plasma DNA non-invasively by combined application of targeted capture sequencing, a bioinformatics process developed for low frequency mutation detection, and a strict variant interpretation strategy. This method has the potential to facilitate clinical diagnosis and to increase diagnosis accuracy, particularly when the phenotype is not clear enough to make a differential diagnosis. Because de novo mutations have been related to many birth defects, this method can also be applied to other diseases. This method also has the potential to be used as a prenatal screening assay in early gestation weeks; the detection of pathogenic variants may indicate the need for more detailed ultrasound detection or further invasive prenatal diagnosis, which would be useful in areas where access to professional medical services is difficult. However, notably, a further study involving a larger sample size is needed to determine the sensitivity and specificity in different foetal fractions. The increased sample size will be helpful in establishing an optimized pipeline that can maximize the real positive while minimize the false positive results.

## Supporting Information

S1 Fig The size distribution of foetal DNA and total DNA. (A) case 1. (B) control case 2. Blue line: the size distribution of foetal DNA; red line: the size distribution of total DNA.
(TIFF)

S2 Fig The ultrasound result of case 1. Ultrasound examinations revealed a single live foetus with a biparietal diameter of 7.8 cm (26 weeks, 3 days equivalent) and an amniotic fluid index of 28 (polyhydramnios). The head circumference measured 27.6 cm; femur length, 2.4 cm; abdomen circumference, 22.8 cm; and humerus, 2.2 cm.
(TIF)

S3 Fig The ultrasound result of case 2. Ultrasound examinations revealed a breech, single live foetus with a biparietal diameter of 5.2 cm (18 weeks, 3 days equivalent) and an amniotic fluid index of 6.4 (polyhydramnios). The head circumference measured 18.3 cm; femur length, 2.1 cm; and abdomen circumference, 16.7 cm.
(TIF)

S4 Fig The ultrasound result of case 3. Ultrasound examinations revealed a single live foetus with a biparietal diameter of 7.5 cm and an amniotic fluid index of 23.5. The head circumference measured 26.5 cm; femur length, 3.8 cm; and abdomen circumference, 23.1 cm.
(TIF)

S5 Fig Distribution of allele frequency of non-reference alleles under different conditions. (A) Case 1. (B) Case 2. (C) Case 3. (D) Control case 1. (E) Control case 2. Hom-ref: alleles that were heterozygous in the mother, while the foetus was homozygous for the reference allele; het: alleles that were heterozygous in both the mother and the foetus; hom-alt: allele that was heterozygous in mother, while the foetus was homozygous for the non-reference allele.
(TIF)

S1 Table Mean depth of targeted region in chromosome Y. (DOC)

S2 Table Detailed information of foetal specific variants detected in plasma DNA in case 1. (XLS)

S3 Table Detailed information of foetal specific variants detected in plasma DNA in case 2. (XLS)

S4 Table Detailed information of foetal specific variants detected in plasma DNA in case 3. (XLS)

S5 Table Detailed information of foetal specific variants detected in plasma DNA in control case 1. (XLS)

S6 Table Detailed information of foetal specific variants detected in plasma DNA in control case 2. (XLS)

S7 Table Clinical information of the three families whose foetuses were observed to have skeletal abnormalities. (DOC)

S8 Table Annotation of variants detected in gene of interest in plasma sample of case 1. (XLSX)

S9 Table Annotation of variants detected in gene of interest in plasma sample of case 2. (XLS)

S10 Table Annotation of variants detected in gene of interest in plasma sample of case 3. (XLS)

S11 Table Annotation of variants detected in gene of interest in plasma sample of control case 1. (XLS)

S12 Table Annotation of variants detected in gene of interest in plasma sample of control case 2. (XLS)

S13 Table Statistics of reads supporting c.742**C>T** variant in *FGFR3* in each sample. (DOC)

S14 Table Statistics of reads supporting c.1774**G>A** variant in *COL1A2* in each sample. (DOC)

S15 Table Statistics of reads supporting c.1138**G>A** variant in *FGFR3* in each sample. (DOC)

S16 Table Performance of SNV detection in plasma samples without the use of duplication information. (DOC)
