# Supplementary figures and images for: Non-Invasive Prenatal Diagnosis of Lethal Skeletal Dysplasia by Targeted Capture Sequencing of Maternal Plasma
Source: PLoS One. 2016 Jul 19;11(7):e0159355. doi: 10.1371/journal.pone.0159355 (PMC4959253; doi:10.1371/journal.pone.0159355)

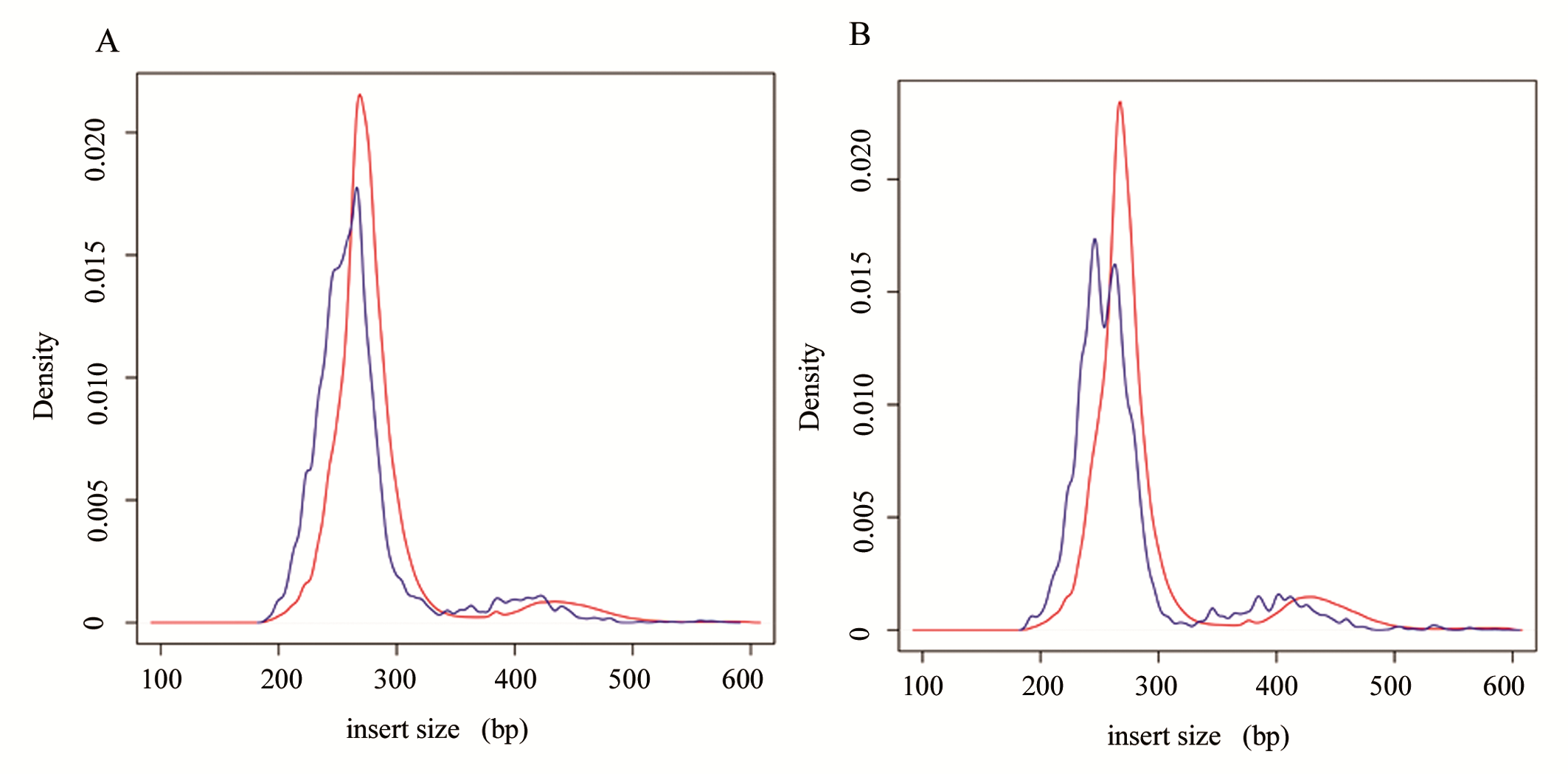

Supplement: S1 Fig — (A) case 1. (B) control case 2. Blue line: the size distribution of foetal DNA; red line: the size distribution of total DNA. (TIFF) [file pone.0159355.s001.tiff]

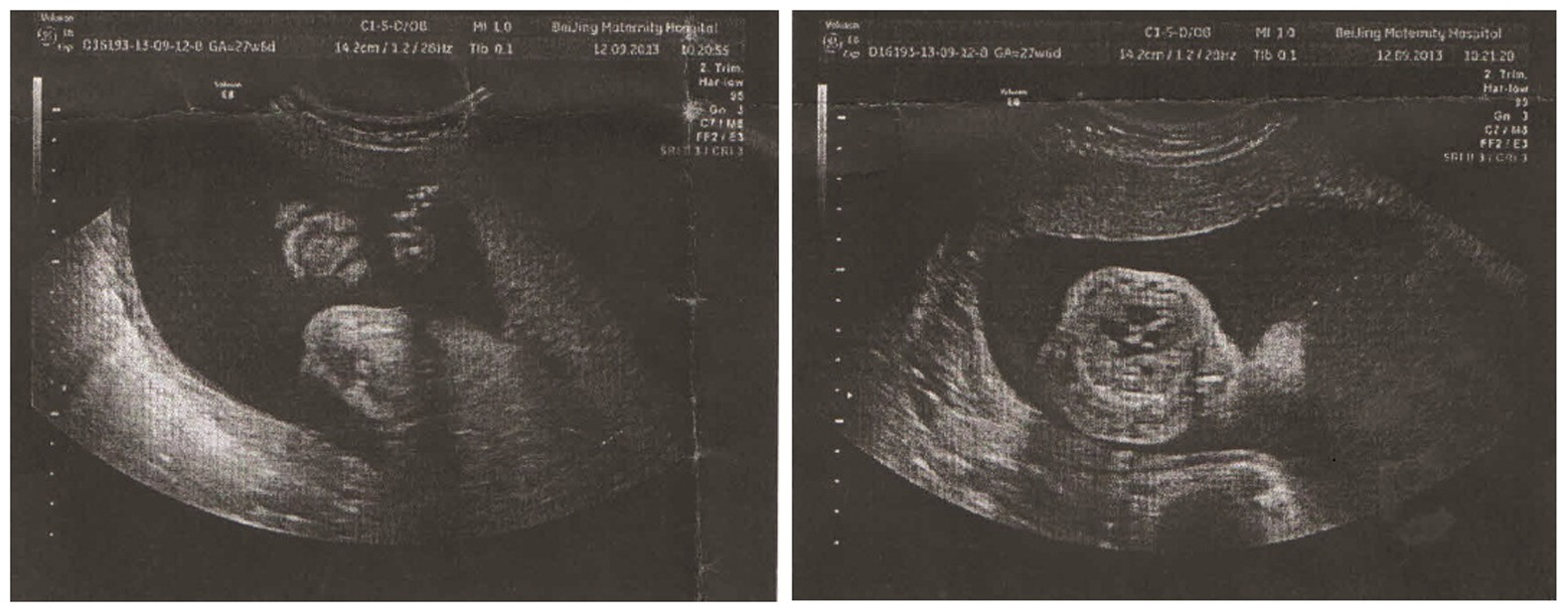

Supplement: S2 Fig — Ultrasound examinations revealed a single live foetus with a biparietal diameter of 7.8 cm (26 weeks, 3 days equivalent) and an amniotic fluid index of 28 (polyhydramnios). The head circumference measured 27.6 cm; femur length, 2.4 cm; abdomen circumference, 22.8 cm; and humerus, 2.2 cm. (TIF) [file pone.0159355.s002.tif]

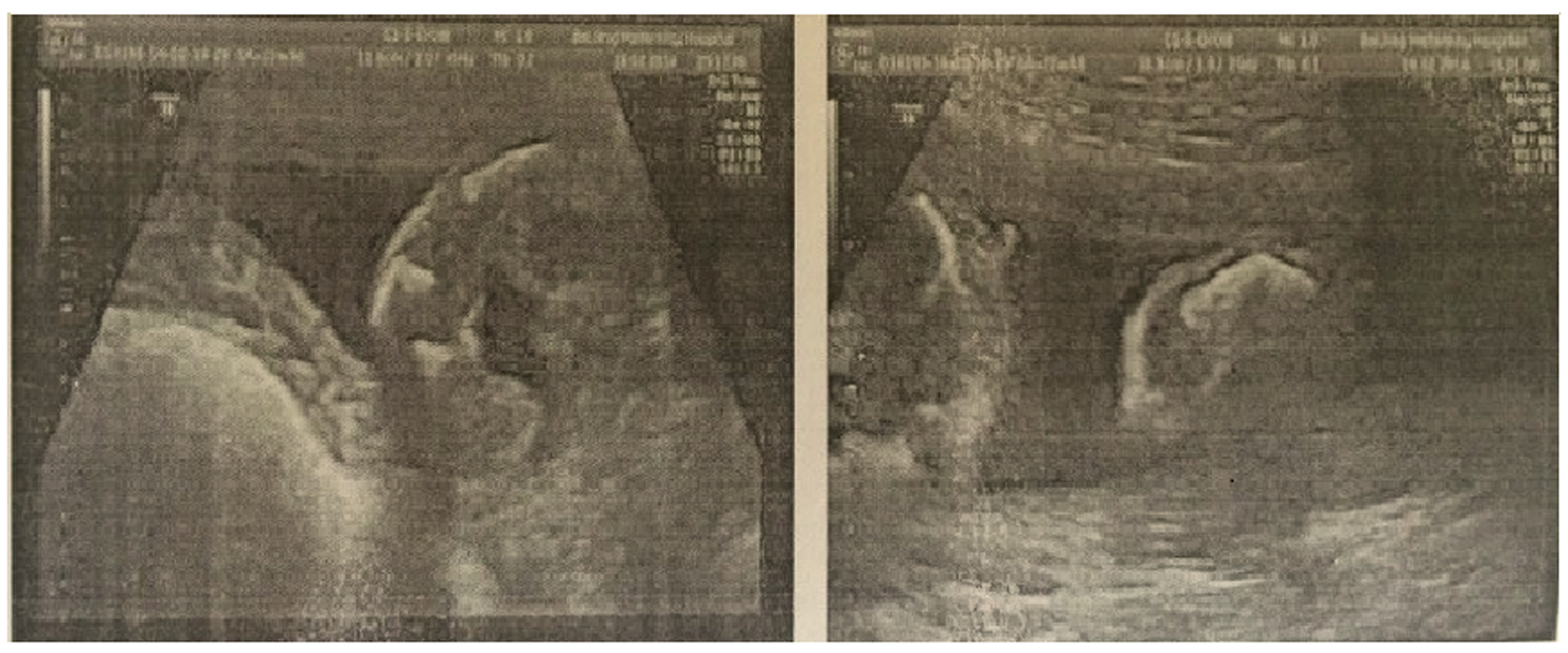

Supplement: S3 Fig — Ultrasound examinations revealed a breech, single live foetus with a biparietal diameter of 5.2 cm (18 weeks, 3 days equivalent) and an amniotic fluid index of 6.4 (polyhydramnios). The head circumference measured 18.3 cm; femur length, 2.1 cm; and abdomen circumference, 16.7 cm. (TIF) [file pone.0159355.s003.tif]

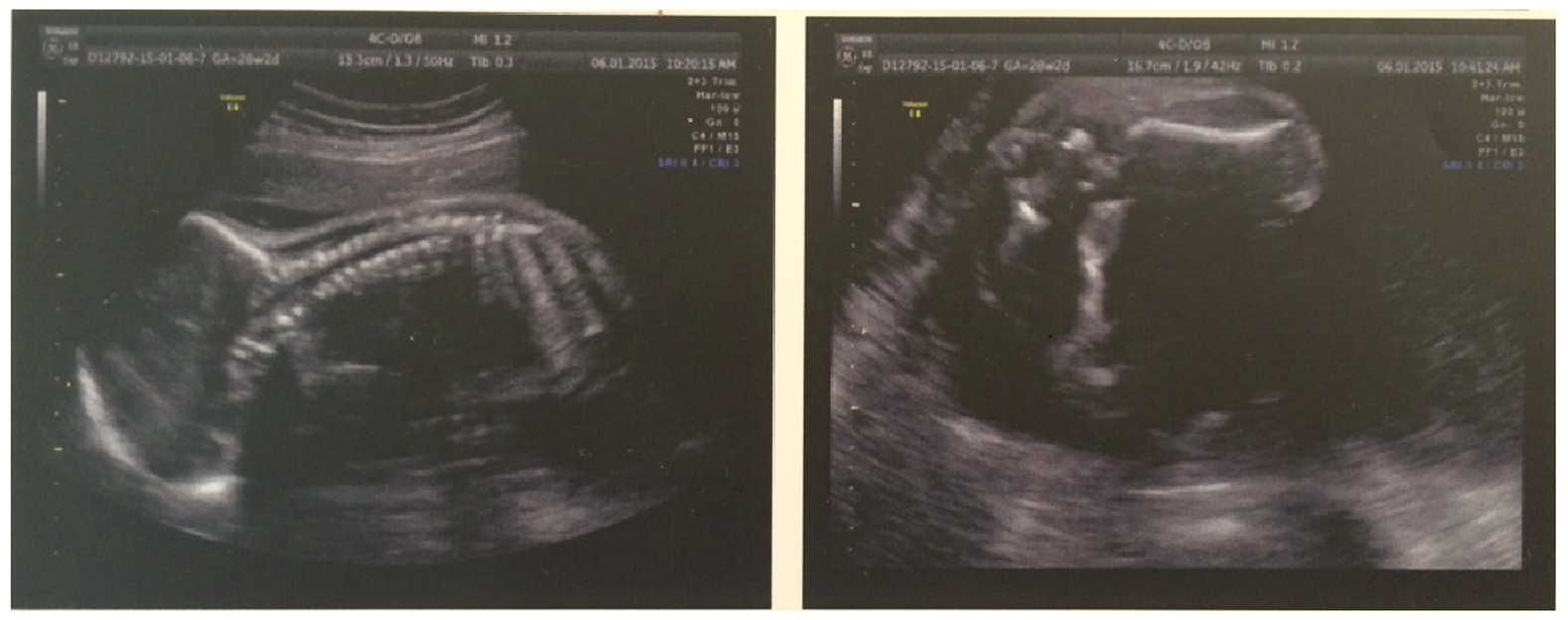

Supplement: S4 Fig — Ultrasound examinations revealed a single live foetus with a biparietal diameter of 7.5 cm and an amniotic fluid index of 23.5. The head circumference measured 26.5 cm; femur length, 3.8 cm; and abdomen circumference, 23.1 cm. (TIF) [file pone.0159355.s004.tif]

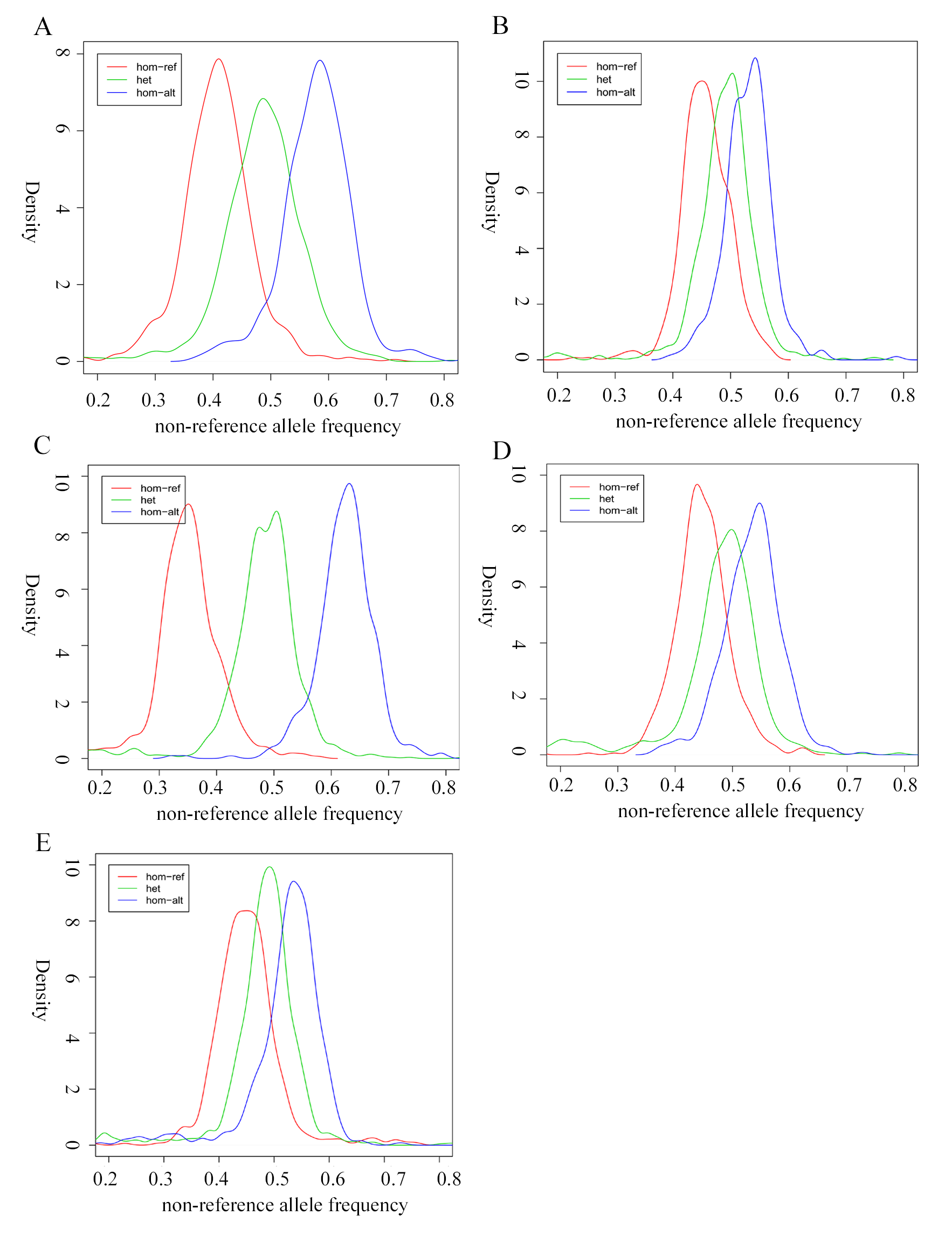

Supplement: S5 Fig — (A) Case 1. (B) Case 2. (C) Case 3. (D) Control case 1. (E) Control case 2. Hom-ref: alleles that were heterozygous in the mother, while the foetus was homozygous for the reference allele; het: alleles that were heterozygous in both the mother and the foetus; hom-alt: allele that was heterozygous in mother, while the foetus was homozygous for the non-reference allele. (TIF) [file pone.0159355.s005.tif]
