# Supplementary material for: Non-Invasive Prenatal Diagnosis of Lethal Skeletal Dysplasia by Targeted Capture Sequencing of Maternal Plasma
Source: PLoS One. 2016 Jul 19;11(7):e0159355. doi: 10.1371/journal.pone.0159355 (PMC4959253; doi:10.1371/journal.pone.0159355)
Supplement: S1 Table — (DOC) [file pone.0159355.s006.doc]

**Table S1 mean depth of targeted region in chromosome Y**

| **Sample** | **Mean depth of targeted region in chromosome Y** |
| --- | --- |
| Case1 plasma sample | 17.97 |
| Case2 plasma sample | 1.18 |
| Case3 plasma sample | 0.48 |
| Control case1 plasma sample | 0.54 |
| Control case2 plasma sample | 21.92 |
