# Supplementary material for: Non-Invasive Prenatal Diagnosis of Lethal Skeletal Dysplasia by Targeted Capture Sequencing of Maternal Plasma
Source: PLoS One. 2016 Jul 19;11(7):e0159355. doi: 10.1371/journal.pone.0159355 (PMC4959253; doi:10.1371/journal.pone.0159355)
Supplement: S7 Table — (DOC) [file pone.0159355.s012.doc]

**Table S7 clinical information of studied families**

| **Family** | **Maternal age** | **the history of pregancy** | **Gestation period of ultrasound diagnosis** | **Invasive prenatal diagnosis** |
| --- | --- | --- | --- | --- |
| Case 1 | 26 | gravida 2, para 0 | 27 wk 6d | amniocentesis |
| Case 2 | 29 | gravida 1, para 1 | 22 wk 3d | amniocentesis |
| Case 3 | 29 | gravida 0, para 0 | 28wk 2d | amniocentesis |
