# Supplementary material for: Non-Invasive Prenatal Diagnosis of Lethal Skeletal Dysplasia by Targeted Capture Sequencing of Maternal Plasma
Source: PLoS One. 2016 Jul 19;11(7):e0159355. doi: 10.1371/journal.pone.0159355 (PMC4959253; doi:10.1371/journal.pone.0159355)
Supplement: S13 Table — (DOC) [file pone.0159355.s018.doc]

**Table S13** Statistics of reads supporting c.742C>T variant in FGFR3 in each sample

| **family** | **sample** | **After the filter of duplication reads** | | | **Before the filter of duplication reads** | | |
| --- | --- | --- | --- | --- | --- | --- | --- |
|  |  | **depth** | **Variant Allele ratio** | **Variant reads number** | **depth** | **Variant Allele ratio** | **Variant reads number** |
| Case 1 | fetus | 35 | 0.40 | 14 | 43 | 0.40 | 17 |
|  | mother | 24 | 0.00 | 0 | 28 | 0.00 | 0 |
|  | father | 27 | 0.00 | 0 | 33 | 0.00 | 0 |
|  | plasma | 69 | 0.20 | 14 | 144 | 0.22 | 31 |
| Case 2 | fetus | 15 | 0.00 | 0 | 15 | 0.00 | 0 |
|  | mother | 15 | 0.00 | 0 | 15 | 0.00 | 0 |
|  | father | 19 | 0.00 | 0 | 21 | 0.00 | 0 |
|  | plasma | 73 | 0.00 | 0 | 129 | 0.00 | 0 |
| Case 3 | fetus | 17 | 0.00 | 0 | 22 | 0.00 | 0 |
|  | mother | 26 | 0.00 | 0 | 36 | 0.00 | 0 |
|  | father | 31 | 0.00 | 0 | 33 | 0.00 | 0 |
|  | plasma | 45 | 0.00 | 0 | 58 | 0.00 | 0 |
| Control case 1 | fetus | 36 | 0.00 | 0 | 40 | 0.00 | 0 |
|  | mother | 23 | 0.00 | 0 | 28 | 0.00 | 0 |
|  | father | 47 | 0.02 | 1 | 59 | 0.02 | 1 |
|  | plasma | 55 | 0.00 | 0 | 77 | 0.00 | 0 |
| Control case 2 | fetus | 37 | 0.00 | 0 | 40 | 0.00 | 0 |
|  | mother | 11 | 0.00 | 0 | 13 | 0.00 | 0 |
|  | father | 29 | 0.00 | 0 | 33 | 0.00 | 0 |
|  | plasma | 78 | 0.01 | 1 | 124 | 0.01 | 1 |
