# Supplementary material for: Non-Invasive Prenatal Diagnosis of Lethal Skeletal Dysplasia by Targeted Capture Sequencing of Maternal Plasma
Source: PLoS One. 2016 Jul 19;11(7):e0159355. doi: 10.1371/journal.pone.0159355 (PMC4959253; doi:10.1371/journal.pone.0159355)
Supplement: S14 Table — (DOC) [file pone.0159355.s019.doc]

**Table S14 Statistics of reads supporting c.1774G>A variant in COL1A2 in each sample**

| **family** | **sample** | **After the filter of duplication reads** | | | **Before the filter of duplication reads** | | |
| --- | --- | --- | --- | --- | --- | --- | --- |
|  |  | **depth** | **Variant Allele ratio** | **Variant reads number** | **depth** | **Variant Allele ratio** | **Variant reads number** |
| Case1 | fetus | 357 | 0.01 | 2 | 459 | 0.00 | 2 |
|  | mother | 268 | 0.00 | 0 | 333 | 0.00 | 0 |
|  | father | 301 | 0.00 | 0 | 362 | 0.00 | 0 |
|  | plasma | 167 | 0.00 | 0 | 1550 | 0.00 | 0 |
| Case2 | fetus | 208 | 0.48 | 100 | 243 | 0.47 | 115 |
|  | mother | 488 | 0.00 | 1 | 544 | 0.00 | 1 |
|  | father | 270 | 0.00 | 0 | 328 | 0.00 | 0 |
|  | plasma | 311 | 0.05 | 16 | 2504 | 0.06 | 138 |
| Case3 | fetus | 292 | 0.00 | 0 | 370 | 0.00 | 0 |
|  | mother | 348 | 0.00 | 0 | 440 | 0.00 | 0 |
|  | father | 310 | 0.00 | 0 | 406 | 0.00 | 0 |
|  | plasma | 473 | 0.00 | 1 | 1150 | 0.00 | 2 |
| Control case 1 | fetus | 501 | 0.00 | 1 | 682 | 0.00 | 1 |
|  | mother | 328 | 0.00 | 1 | 407 | 0.00 | 1 |
|  | father | 493 | 0.00 | 1 | 668 | 0.00 | 1 |
|  | plasma | 534 | 0.00 | 1 | 1629 | 0.00 | 1 |
| Control case 2 | fetus | 486 | 0.00 | 0 | 641 | 0.00 | 0 |
|  | mother | 278 | 0.00 | 0 | 360 | 0.00 | 0 |
|  | father | 426 | 0.00 | 0 | 549 | 0.00 | 0 |
|  | plasma | 372 | 0.00 | 0 | 1636 | 0.00 | 1 |
