# Supplementary material for: Non-Invasive Prenatal Diagnosis of Lethal Skeletal Dysplasia by Targeted Capture Sequencing of Maternal Plasma
Source: PLoS One. 2016 Jul 19;11(7):e0159355. doi: 10.1371/journal.pone.0159355 (PMC4959253; doi:10.1371/journal.pone.0159355)
Supplement: S15 Table — (DOC) [file pone.0159355.s020.doc]

**Table S15 Statistics of reads supporting c.1138G>A variant in FGFR3 in each sample**

| **family** | **sample** | **After the filter of duplication reads** | | | **Before the filter of duplication reads** | | |
| --- | --- | --- | --- | --- | --- | --- | --- |
|  |  | **depth** | **Variant Allele ratio** | **Variant reads number** | **depth** | **Variant Allele ratio** | **Variant reads number** |
| Case 1 | fetus | 174 | 0.00 | 0 | 207 | 0.00 | 0 |
|  | mother | 183 | 0.00 | 0 | 218 | 0.00 | 0 |
|  | father | 158 | 0.00 | 0 | 182 | 0.00 | 0 |
|  | plasma | 182 | 0.00 | 0 | 1367 | 0.00 | 2 |
| Case 2 | fetus | 156 | 0.00 | 0 | 169 | 0.00 | 0 |
|  | mother | 160 | 0.00 | 0 | 171 | 0.00 | 0 |
|  | father | 106 | 0.00 | 0 | 128 | 0.00 | 0 |
|  | plasma | 375 | 0.00 | 0 | 1840 | 0.00 | 1 |
| Case 3 | fetus | 155 | 0.50 | 78 | 182 | 0.51 | 92 |
|  | mother | 173 | 0.00 | 0 | 198 | 0.00 | 0 |
|  | father | 158 | 0.00 | 0 | 188 | 0.01 | 1 |
|  | plasma | 391 | 0.14 | 56 | 734 | 0.15 | 111 |
| Control case 1 | fetus | 373 | 0.00 | 1 | 469 | 0.00 | 1 |
|  | mother | 121 | 0.00 | 0 | 136 | 0.00 | 0 |
|  | father | 319 | 0.00 | 1 | 413 | 0.00 | 2 |
|  | plasma | 523 | 0.00 | 1 | 1208 | 0.00 | 2 |
| Control case 2 | fetus | 269 | 0.00 | 0 | 306 | 0.00 | 0 |
|  | mother | 135 | 0.00 | 0 | 168 | 0.00 | 0 |
|  | father | 187 | 0.00 | 0 | 235 | 0.00 | 0 |
|  | plasma | 403 | 0.00 | 0 | 1175 | 0.00 | 0 |
