# Supplementary material for: Non-Invasive Prenatal Diagnosis of Lethal Skeletal Dysplasia by Targeted Capture Sequencing of Maternal Plasma
Source: PLoS One. 2016 Jul 19;11(7):e0159355. doi: 10.1371/journal.pone.0159355 (PMC4959253; doi:10.1371/journal.pone.0159355)
Supplement: S16 Table — (DOC) [file pone.0159355.s021.doc]

**Table S16 Performance of SNV detection in plasma samples without the use of duplication information**

|  | **Fetal conc** | **Numbers of FN** | **Numbers of FP** | **Numbers of TP** | **TPR** | **PPV** |
| --- | --- | --- | --- | --- | --- | --- |
| Case 2 | 6.84% | 79 | 173 | 77 | 92.41% | 29.67% |
| Control case 1 | 9.49% | 114 | 104 | 112 | 98.26% | 52.07% |
| Control case 2 | 10.37% | 115 | 83 | 114 | 98.26% | 57.65% |
| Case 1 | 20.76% | 112 | 56 | 109 | 97.32% | 66.06% |
| Case 3 | 30.56% | 105 | 82 | 108 | 100.00% | 56.15% |

Fetal conc: the fraction of fetal DNA; FN: the number of false negative loci, allele that is detected in fetal gDNA sample but is not present in plasma sample; FP: the number of false positive loci, allele that is not detected in fetal gDNA sample but is present in plasma sample; TP:the number of loci shared by plasma and fetal gDNA.
